# Expression of Pentose Phosphate Pathway-Related Proteins in Breast Cancer

**DOI:** 10.1155/2018/9369358

**Published:** 2018-02-25

**Authors:** Junjeong Choi, Eun-Sol Kim, Ja Seung Koo

**Affiliations:** ^1^College of Pharmacy, Yonsei Institute of Pharmaceutical Sciences, Yonsei University, Incheon, Republic of Korea; ^2^Department of Pathology, Yonsei University College of Medicine, Seoul, Republic of Korea

## Abstract

**Purpose:**

The purpose of this study was to assess the expression of pentose phosphate pathway- (PPP-) related proteins and their significance in clinicopathologic factors of breast cancer.

**Methods:**

Immunohistochemical staining for PPP-related proteins (glucose-6-phosphate dehydrogenase [G6PDH], 6-phosphogluconolactonase [6PGL], 6-phosphogluconate dehydrogenase [6PGDH], and nuclear factor-erythroid 2-related factor 2 [NRF2]) was performed using tissue microarray (TMA) of 348 breast cancers. mRNA levels of these markers in publicly available data from the Cancer Genome Atlas project and Kaplan-Meier plotters were analyzed.

**Results:**

Expression of G6PDH and 6PGL was higher in HER-2 type (*p* < 0.001 and *p* = 0.009, resp.) and lower in luminal A type. 6PGDH expression was detected only in TNBC subtype (*p* < 0.001). G6PDH positivity was associated with ER negativity (*p* = 0.001), PR negativity (*p* = 0.001), and HER-2 positivity (*p* < 0.001), whereas 6PGL positivity was associated with higher T stage (*p* = 0.004). The 562 expression profile from the TCGA database revealed increased expression of G6PDH and 6PG in the tumor compared with normal adjacent breast tissue. The expression of G6PDH was highest in HER-2 type. HER-2 and basal-like subtypes showed higher expression of 6PGDH than luminal types.

**Conclusion:**

PPP-related proteins are differentially expressed in breast cancer according to molecular subtype, and higher expression of G6PDH and 6PGL was noted in HER-2 subtype.

## 1. Introduction

The pentose phosphate pathway (PPP) is a major glucose catabolic pathway parallel to glycolysis that is responsible for synthesis of the nucleotide precursor ribose and nicotinamide adenine dinucleotide phosphate (NADPH) required for glucose metabolism. The PPP consists of an oxidative branch and a nonoxidative branch. Glucose-6-phosphate dehydrogenase (G6PDH), 6-phosphogluconolactonase (6PGL), and 6-phosphogluconate dehydrogenase (6PGDH) are major proteins involved in the synthesis of NADPH and ribonucleotide through the oxidative branch. The PPP provides pentose phosphate required for nucleic acid synthesis in rapidly growing cells. In cancers, the PPP supplies not only pentose phosphate but also NADPH, which is important for lipid synthesis and cell survival under stressful circumstances. Thus, the importance of the PPP is highlighted in rapidly growing cancer cells, and previous studies have reported that the expression of PPP-related enzymes is increased in human cancer tissues [1–3].

As breast cancer is a heterogeneous disease in terms of clinical, histologic, and molecular genetic aspects, a lot of effort has focused on classifying the disease into subgroups with similar characteristics. The molecular genetic subtypes of luminal A, luminal B, HER-2, and normal breast-like and basal-like type were identified by gene profiling analysis [4–6]. Separately, breast cancers with a combined negative expression of estrogen receptor (ER), progesterone receptor (PR), and human epidermal growth factor receptor 2 (HER-2), which has therapeutic implications, are classified as triple-negative breast cancer (TNBC) [7]. This intrinsic heterogeneity in the molecular genetics of breast cancer gives rise to heterogeneity in histology, clinical features, treatment response, and prognosis as well as metabolic features. Previous studies have reported increased expression of glycolysis-related proteins GLUT-1 and CAIX in the basal-like type and TNBC [8, 9] and increased expression of glutaminolysis-related proteins in the HER-2 type [10], suggesting a relationship between metabolism and molecular subtypes of breast cancer. However, there are a limited number of studies on the expression of pentose phosphate pathway-related proteins in breast cancer. The objective of this study was to assess the expression of pentose phosphate pathway-related proteins according to breast cancer molecular subtype and their biological and clinical implications.

## 2. Materials and Methods

### 2.1. Patient Selection and Histologic Evaluation

Breast cancer tissues were obtained form 348 patients, who were diagnosed as invasive ductal carcinoma not otherwise specified and had undergone mastectomy at Severance Hospital (Seoul, Republic of Korea) during the period of January 2001 to December 2006. This study group is selected form previously published study population [11]. Exclusion criteria were preoperative chemotherapy or hormonal therapy.

This study was approved by the Institutional Review Board of Yonsei University Severance Hospital. The IRB waived the need for informed consent from patients. Breast pathologist (Ja Seung, Koo) reviewed the histologic features by using hematoxylin & eosin- (H&E-) stained slides for all cases. Histologic grade was assessed using the Nottingham grading system [12]. Clinicopathologic parameters including patient age at initial diagnosis, lymph node metastasis, tumor recurrence, distant metastasis, were retrieved from electronic medical records of each case.

### 2.2. Tissue Microarray

A TMA construct of 3 mm diameter cores was generated from the 10% neutrally buffered formalin-fixed, paraffin-embedded tissue blocks of radical prostatectomy specimens using a tissue microarrayer. Two representative cores from different cancer areas were included for each case. Each tissue core was assigned a unique tissue microarray location number that was linked to a database containing deidentified clinicopathologic data.

### 2.3. Immunohistochemistry

Antibodies used for immunohistochemistry are summarized in Table 1. Briefly, representative paraffin blocks were cut consecutively at 4 *μ*m thickness, and sections were deparaffinized in xylene and treated with 0.3% hydrogen peroxide in methanol for 20 minutes to block any endogenous peroxidase activity. Citrate buffer was used for antigen retrieval. Nonspecific binding was limited by using protein blocking buffer for 10 minutes. The sections were washed in phosphate-buffered saline and then incubated with the primary antibody for 20 minutes at room temperature. The samples were then incubated in secondary antibody (biotinylated) for 10 minutes, followed by incubation with streptavidin-horseradish peroxidase for 10 minutes, and exposed to diaminobenzidine, which was used as a chromogen. All labeled streptavidin-biotin-horseradish peroxidase system chemicals were obtained from Dako Cytomation Corp. (Carpinteria, CA, USA). Counterstaining was performed with Mayer's hematoxylin. Negative controls were treated similarly with the exception of incubation with the primary antibody (nonspecific staining control).

### 2.4. Interpretation of Immunohistochemical Staining

All immunohistochemical markers were evaluated twice by two independent investigators blinded to the clinical details. Cutoff value of 1% or more positively stained nuclei was employed to define ER and PR positivity [13]. HER-2 status was analyzed according to the American Society of Clinical Oncology (ASCO)/College of American Pathologists (CAP) guidelines using the following categories: 0 = no immunostaining; 1+ = weak incomplete membranous staining, less than 10% of tumor cells; 2+ = complete membranous staining, either uniform or weak in at least 10% of tumor cells; and 3+ = uniform intense membranous staining in at least 30% of tumor cells [14]. HER-2 immunostaining was considered positive when strong (3+) membranous staining was observed, whereas cases with 0 to 1+ staining were counted as negative. Cases showing 2+ HER-2 expression were further evaluated for HER-2 amplification by fluorescent in situ hybridization (FISH).

Immunohistochemical markers for G6PDH, 6PGL, 6PGDH, and NRF2 were assessed by a semiquantitative evaluation method as follows [15]: 0: negative or weak immunostaining in <1% of the tumor; 1: focal expression in 1–10% of the tumor; 2: positive in 11%–50% of the tumor; and 3: positive in 51%–100% of the tumor. The evaluation was made for the whole area of the tumor, and cases with a score greater than 2 were classified as positive.

### 2.5. Tumor Phenotype Classification

We classified each case breast cancer into molecular phenotype by surrogate immunohistochemistry results for ER, PR, HER-2, Ki-67 labeling index (LI), and FISH results for HER-2 as follows [16]: *luminal A type*, ER or/and PR positive, HER-2 negative, and Ki-67 LI < 14%; *luminal B type* (*HER-2 negative*), ER or/and PR positive, HER-2 negative, and Ki-67 LI ≥ 14%; *luminal B type* (*HER-2 positive*), ER or/and PR positive and HER-2 overexpressed or/and amplified; *HER-2 overexpression type*, ER and PR negative and HER-2 overexpressed or/and amplified; and *TNBC type*: ER, PR, and HER-2 negative.

### 2.6. Validation of Expression of PPP-Related Markers in a Public Database

We obtained clinical information and level 3 normalized gene expression (RSEM) values from RNA sequencing data of breast cancer (BRCA) from the Broad Genome Data Analysis Center (GDAC) Firehose server (version 01-28-2016) and filtered out genes with an expression equal to zero in more than 50% of samples. PAM50 classification information and patient survival information were also retrieved from the GDAC. We normalized the value again with the voom function of the limma package using R software (R version 3.3.1.). Another set of survival analyses was performed using the Kaplan-Meier plotter [17].

### 2.7. Statistical Analysis

Statistical analyses were performed with SPSS for Windows, Version 23.0 (SPSS Inc., Chicago, IL, USA). Student's *t*-test, and Fisher's exact tests were employed for continuous and categorical variablefor statistical significance. In case of multiple comparisons, an adjusted *p* value with application of the Bonferroni multiple comparison procedure was used. *p* value < 0.05 was considered to be statistically significant. Kaplan-Meier survival curves and log-rank statistics were employed to evaluate time to tumor recurrence and overall survival. Multivariate regression analysis was performed using the Cox proportional hazard model.

## 3. Results

### 3.1. Basal Characteristics of Breast Cancer

The 348 subjects of this study comprised 162 luminal A (46.6%), 84 luminal B (24.1), 27 HER-2 type (7.6%), and 75 TNBC (21.6%) subtypes. TNBC showed higher histologic grade (*p* < 0.001) and higher Ki-67 LI (*p* < 0.001) (Table 2).

### 3.2. Expression of Pentose Phosphate Pathway-Related Proteins in Breast Cancer

**Figure 1:**
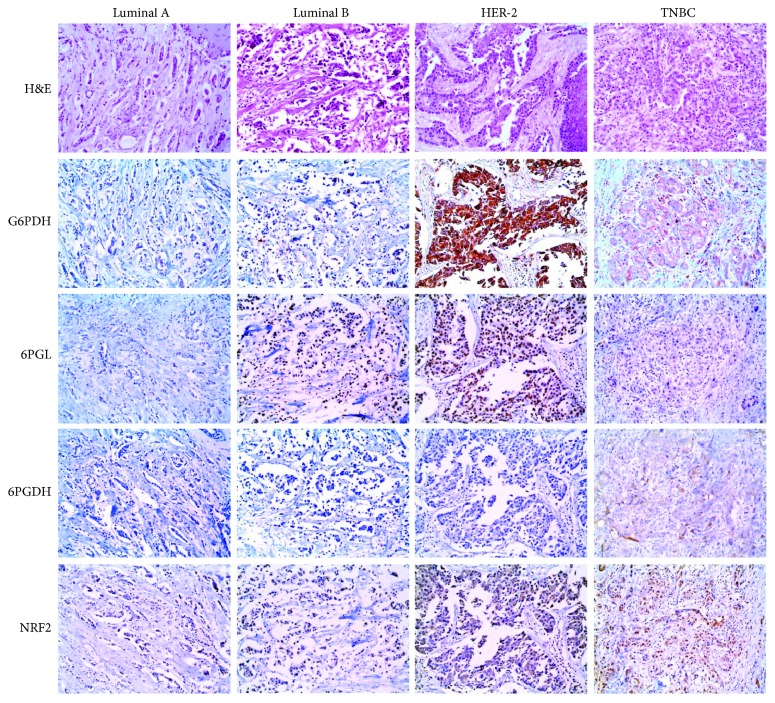
Expression of pentose phosphate pathway-related proteins in breast cancer. G6PDH and 6PGL show higher expression in HER-2 type and lower expression in luminal A type. 6PGDH expression is detected only in the TNBC subtype.

The expression of pentose phosphate pathway-related proteins was assessed according to the molecular subtypes of breast cancer (Figure 1). Expression of G6PDH (*p* < 0.001), 6PGL (*p* = 0.009), and 6PGDH (*p* < 0.001) was identified; G6PDH and 6PGL showed higher expression in the HER-2 type and lower expression in luminal A type. Expression of 6PGDH was detected only in the TNBC subtype (Table 3).

### 3.3. Correlation between Expression of Pentose Phosphate Pathway-Related Proteins and Clinicopathologic Factors

**Figure 2:**
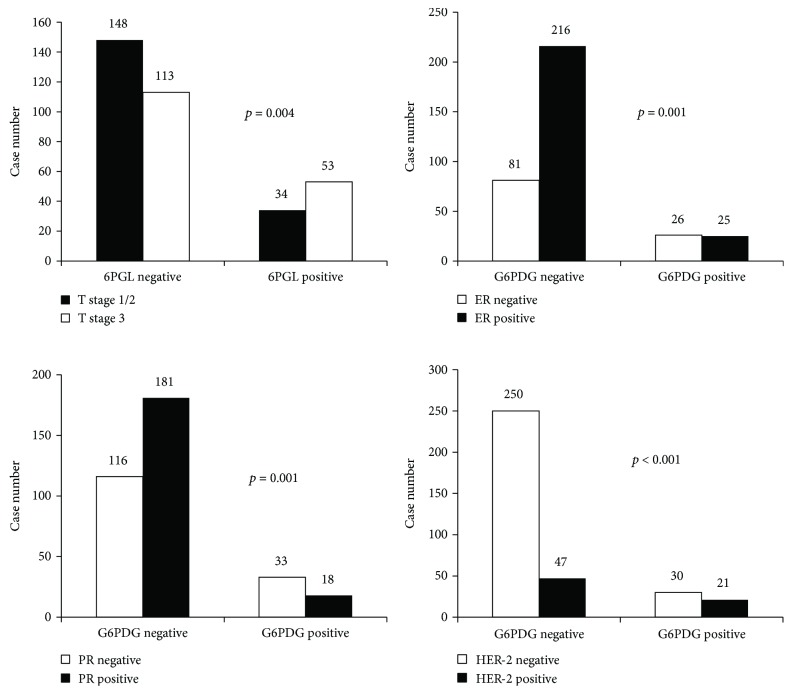
Correlation between expression of pentose phosphate pathway-related proteins and clinicopathologic factors. G6PDH positivity is associated with ER negativity (*p* = 0.001), PR negativity (*p* = 0.001), and HER-2 positivity (*p* < 0.001), and 6PGL positivity is associated with higher T stage (*p* = 0.004).

The expression of pentose phosphate pathway-related proteins and clinicopathologic parameters was assessed (Figure 2). G6PDG positivity was associated with ER negativity (*p* = 0.001), PR negativity (*p* = 0.001), and HER-2 positivity (*p* < 0.001), and 6PGL positivity was associated with higher T stage (*p* = 0.004).

### 3.4. The Impact of Expression of Pentose Phosphate Pathway-Related Proteins on Patient Prognosis

Univariate analysis of patient survival did not show statistically significant differences with regard to expression of pentose phosphate pathway-related proteins (Table 4).

### 3.5. Validation of Expression of PPP-Related Markers in a Public Database

**Figure 3:**
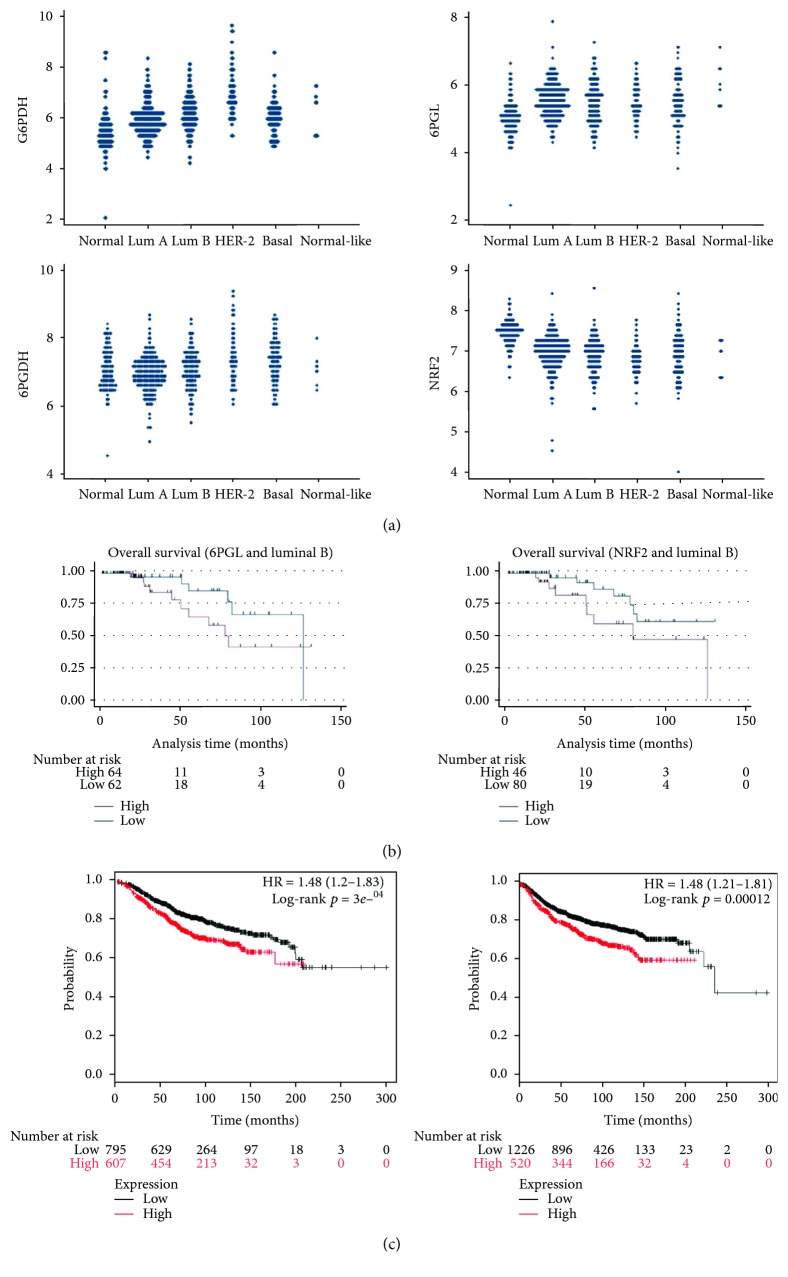
Validation of expression of pentose phosphate pathway-related proteins in a TCGA cohort and Kaplan-Meier plotter. (a). Expression of pentose phosphate pathway-related proteins in a TCGA cohort of breast cancer shows increased expression of G6PDH and 6PGDH. (b). There was a tendency toward longer overall survival with lower expression of 6PGL and NRF2 in the luminal B subtype (*p* = 0.109 and *p* = 0.065) (c). Higher expression of G6PDH is associated with longer overall survival (left) and disease metastasis-free survival (right) in a Kaplan-Meier plotter cohort.

A total of 526 cases were retrieved from the TCGA study. After normalization, the mRNA level of each marker was assessed. G6PDH expression was generally increased in the tumor compared with normal adjacent breast tissue, and expression was highest in the HER-2 type compared with other types. 6PGL expression was generally increased in tumors compared with adjacent normal cells; however, differential expression among tumor subtypes was not identified. Higher 6PGDH expression compared to normal tissue was noted in HER-2 and basal-like groups. The HER-2 type and basal-like subtype showed higher expression of 6PGDH than luminal types of tumor. Finally, the expression level of NRF2 was decreased in all tumor subtypes compared with normal tissue. Univariate analysis of patient survival with regard to expression of G6PDG, 6PGL, 6PGDH, and NRF2 did not show statistical significance although there was a tendency toward longer overall survival with lower expression of 6PGL and NRF2 in luminal B subtype (*p* = 0.1092 and *p* = 0.065, Figure 3(b)). In the cohort of the Kaplan-Meier plotter, high expression of G6PD was generally related to longer overall survival and recurrence-free survival (Figure 3(c)).

## 4. Discussion

Malignant tumors generally show a rapid growth rate and invasive traits, which require metabolic remodeling of cancer cells and stromal cells. As such, the pentose phosphate pathway is an indispensable metabolic pathway in malignant tumors because of the demand for a high rate of nucleic acid synthesis during growth and the NADPH necessary for cell survival during oncogenic cellular stress. In addition, reactive oxygen species resulting from the rapidly increased metabolism may trigger mutation of protooncogenes and promote protumorigenic signaling, all of which aggravate oxidative stress in cancer cells. Thus, a mechanism for activating the oxidative PPP is expected to be present in cancer cells for maintenance of a high enough level of NADPH. It is possible that an AMPK-dependent mechanism also exists given that oxidative PPP is dependent on glucose availability.

We assessed the expression of PPP-related proteins in TMAs of human breast cancer tissues and a TCGA data set and both analyses revealed higher expression in HER-2 type cancers and lower expression in luminal type cancers. A previous study revealed activation of the PPP in breast carcinoma cells compared with normal breast tissue [18, 19]; this was confirmed in an analysis of the TCGA data set, which showed increased expression of PPP-related markers in breast cancer tissue compared with normal control.

There are few studies on activation of the PPP in breast cancer. Previous studies on human breast cancer tissue revealed differential expression of proteins related to glycolysis, glutamine metabolism, lipid metabolism, and serine/glycerine metabolism according to molecular subtype [9, 10, 20–22], suggesting unique metabolic properties of each subtype. Generally, subtypes with a high proliferation rate and aggressive biological behaviors, such as HER-2 or TNBC types, show increased expression of metabolic factors [9, 10, 20–22], and comparable results were obtained in this study. A potential mechanism for higher expression of PPP markers in the HER-2 type is the relationship of HER-2 with NRF2. A previous study reported association between NRF2 and HER-2 in the ErbB2/HER-2-positive breast cancer cell line BT-474 [23], with knockdown of NRF2 leading to repression of HER-2 expression. As NRF2 is the key molecule that coordinates the PPP and is known to have a regulatory role in cancer [24], crosstalk between HER-2 and the PPP mediated by NRF2 may exist. A second mechanism involves the fatty acid synthesis pathway in HER-2 type cancer. TP53-mutated breast cancer shows upregulation of PGD, TK, and ribose 5-phosphate isomerase A [25]. Previous studies reported that a high level of transketolase 1 was correlated with HER-2/neu overexpression [26] and that HER-2 overexpression increases the translation of fatty acid synthase and vice versa [27, 28]. Given that NADPH plays a major role in fatty acid synthesis by fatty acid synthase, HER-2 overexpressing tumor cells may use the PPP as a source of NADPH. It is noticeable that resistance to anti-HER-2 therapy can be overcome by blockade of the fatty acid synthesis pathway. Thus, understanding metabolic reprogramming in terms of the PPP seems clinically relevant.

G6PDH is the rate-limiting enzyme in the PPP and is also highlighted in this study of breast cancer. G6PDH reflects oxidative PPP and the equilibrium between glycolysis and the PPP. p53 is a well-known regulator of the PPP through inhibition of the dimerization and activation of G6PDH [29]. This study revealed a difference in the expression of G6PDH among subtypes of breast cancer, and analysis of the TCGA data showed relatively increased G6PDH levels in all subtypes of breast tumor compared with normal breast tissue. Given that expression and activation of G6PDH in cells are tightly regulated, the increased expression of G6PDH in breast cancers may reflect general activation of the PPP. Interestingly, increased expression of G6PDH was most prominent in HER-2 type breast cancers, indicating transcriptionally regulated expression of G6PDH in this specific type of breast cancer.

The role of NRF2 in tumorigenesis is a subject of debate as activation of NRF2 shows both a tumor suppressor role and oncogenic roles [30]. The NRF2 level was downregulated in all types of breast cancers in the TCGA dataset, suggesting a possible role of NRF2 as a tumor suppressor in breast cancer. However, it is also possible that the result of RNA sequencing may not reflect actual activity of NRF2 in cells due to posttranslational regulation by ubiquitination and degradation.

The clinical implication of this study is the identification of the PPP protein as a potential therapeutic target. Previous studies reported that inhibition of PPP proteins resulted in growth inhibition and cell death in leukemia [31], ovary cancer [32], urinary bladder cancer [33], and breast cancer/prostate cancer [34], suggesting that modulation of this pathway may have therapeutic potential in the treatment of cancer.

In conclusion, PPP-related proteins are differentially expressed according to the molecular subtype of breast cancer; in particular, G6PDH and 6PGL are highly expressed in HER-2 type breast cancer. Thus, understanding of the role of this pathway in breast cancers and further studies on the effects of targeting this pathway are needed to clarify the clinical implications of PPP in breast cancers.

## Figures and Tables

**Table 1:** Source, clone, and dilution of antibodies.

Antibody	Company	Reaction site	Clone	Dilution
*Pentose phosphate pathway*-*related proteins*				
G6PDH	Abcam, Cambridge, UK	Cytoplasmic	Polyclonal	1 : 100
6PGL	Abcam, Cambridge, UK	Cytoplasmic and nuclear	ERP1238 (B)	1 : 200
6PGDH	Abcam, Cambridge, UK	Cytoplasmic	Polyclonal	1 : 100
NRF2	Abcam, Cambridge, UK	Cytoplasmic and nuclear	Polyclonal	1 : 50
*Molecular subtype*-*related proteins*				
ER	Thermo Scientific, San Diego, CA, USA	Nuclear	SP1	1 : 100
PR	DAKO, Glostrup, Denmark	Nuclear	PgR	1 : 50
HER-2	DAKO, Glostrup, Denmark	Membranous	Polyclonal	1 : 1500
Ki-67	Abcam, Cambridge, UK	Nuclear	MIB	1 : 1000

**Table 2:** Clinicopathologic characteristics of patients according to breast cancer phenotype.

Parameter	Total (*n* = 348) (%)	Luminal A (*n* = 162) (%)	Luminal B (*n* = 84) (%)	HER-2 (*n* = 27) (%)	TNBC (*n* = 75) (%)	*p* value
Age (years)						**0.299**
≤50	202 (58.0)	94 (58.0)	55 (65.5)	13 (48.1)	40 (53.3)	
>50	146 (42.0)	68 (42.0)	29 (34.5)	14 (51.9)	35 (46.7)	
Histologic grade						**<0.001**
I/II	242 (69.5)	147 (90.7)	53 (63.1)	12 (44.4)	30 (40.0)	
III	106 (30.5)	15 (9.3)	31 (36.9)	15 (55.6)	45 (60.0)	
Tumor stage						**0.068**
T1	182 (52.3)	96 (59.3)	42 (50.0)	13 (48.1)	31 (41.3)	
T2/T3	166 (47.7)	66 (40.7)	42 (50.0)	14 (51.9)	44 (58.7)	
Nodal metastasis						0.676
Absent	208 (59.8)	94 (58.0)	48 (57.1)	17 (63.0)	49 (65.3)	
Present	140 (40.2)	68 (42.0)	36 (42.9)	10 (37.0)	26 (34.7)	
Estrogen receptor status						**<0.001**
Negative	107 (30.7)	2 (1.2)	3 (3.6)	27 (100.0)	75 (100.0)	
Positive	241 (69.3)	160 (98.8)	81 (96.4)	0 (0.0)	0 (0.0)	
Progesterone receptor status						**<0.001**
Negative	149 (42.8)	20 (12.3)	27 (32.1)	27 (100.0)	75 (100.0)	
Positive	199 (57.2)	142 (87.7)	57 (67.9)	0 (0.0)	0 (0.0)	
HER-2 status						**<0.001**
Negative	280 (80.5)	162 (100.0)	43 (51.2)	0 (0.0)	75 (100.0)	
Positive	68 (19.5)	0 (0.0)	41 (48.8)	27 (100.0)	0 (0.0)	
Ki-67 LI (%)						**<0.001**
≤14	213 (61.2)	162 (100.0)	24 (28.6)	13 (48.1)	14 (18.7)	
>14	135 (38.8)	0 (0.0)	60 (71.4)	14 (51.9)	61 (81.3)	

TNBC: triple-negative breast cancer.

**Table 3:** Expression of pentose phosphate pathway-related metabolism-related proteins according to breast cancer subtype.

Parameter	Total (*n* = 348) (%)	Luminal A (*n* = 162) (%)	Luminal B (*n* = 84) (%)	HER-2 (*n* = 27) (%)	TNBC (*n* = 75) (%)	*p* value
G6PDH						**<0.001**
Negative	297 (85.3)	149 (92.0)	70 (83.3)	14 (51.9)	64 (85.3)	
Positive	51 (14.7)	13 (8.0)	14 (16.7)	13 (48.1)	11 (14.7)	
6PGL						**0.009**
Negative	261 (75.0)	133 (82.1)	58 (69.0)	15 (55.6)	55 (73.3)	
Positive	87 (25.0)	29 (17.9)	26 (31.0)	12 (44.4)	20 (26.7)	
6PGDH						**<0.001**
Negative	343 (98.6)	162 (100.0)	84 (100.0)	27 (100.0)	70 (93.3)	
Positive	5 (1.4)	0 (0.0)	0 (0.0)	0 (0.0)	5 (6.7)	
NRF2						**0.894**
Negative	311 (89.4)	145 (89.5)	76 (90.5)	23 (85.2)	67 (89.3)	
Positive	37 (10.6)	17 (10.5)	8 (9.5)	4 (14.8)	8 (10.7)	

**Table 4:** Univariate analysis of the impact of expression of pentose phosphate pathway-related proteins in breast cancers on disease-free survival and overall survival by the log-rank test.

Parameter	Number of patients/recurrence/death	Disease-free survival		Overall survival	
		Mean survival (95% CI) months	*p* value	Mean survival (95% CI) months	*p* value
G6PDH			0.752		0.377
Negative	297/28/28	126 (122–131)		129 (125–133)	
Positive	51/4/7	122 (114–130)		120 (109–131)	
6PGL			0.239		0.569
Negative	261/20/24	129 (124–133)		129 (124–133)	
Positive	87/12/11	116 (109–124)		125 (117–133)	
6PGDH			n/a		n/a
Negative	343/32/35	n/a		n/a	
Positive	5/0/0	n/a		n/a	
NRF2			n/a		n/a
Negative	311/32/35	n/a		n/a	
Positive	37/0/0	n/a		n/a	

## References

[1] Riganti C., Gazzano E., Polimeni M., Aldieri E., Ghigo D. (2012). The pentose phosphate pathway: an antioxidant defense and a crossroad in tumor cell fate. *Free Radical Biology & Medicine*.

[2] Zhang C., Zhang Z., Zhu Y., Qin S. (2014). Glucose-6-phosphate dehydrogenase: a biomarker and potential therapeutic target for cancer. *Anti-Cancer Agents in Medicinal Chemistry*.

[3] Lucarelli G., Galleggiante V., Rutigliano M. (2015). Metabolomic profile of glycolysis and the pentose phosphate pathway identifies the central role of glucose-6-phosphate dehydrogenase in clear cell-renal cell carcinoma. *Oncotarget*.

[4] Kwon J. E., Jung W. H., Koo J. S. (2012). Molecules involved in epithelial-mesenchymal transition and epithelial-stromal interaction in phyllodes tumors: implications for histologic grade and prognosis. *Tumour Biology*.

[5] Perou C. M., Sørlie T., Eisen M. B. (2000). Molecular portraits of human breast tumours. *Nature*.

[6] Sorlie T., Perou C. M., Tibshirani R. (2001). Gene expression patterns of breast carcinomas distinguish tumor subclasses with clinical implications. *Proceedings of the National Academy of Sciences of the United States of America*.

[7] Reis-Filho J. S., Tutt A. N. J. (2008). Triple negative tumours: a critical review. *Histopathology*.

[8] Pinheiro C., Sousa B., Albergaria A. (2011). GLUT1 and CAIX expression profiles in breast cancer correlate with adverse prognostic factors and MCT1 overexpression. *Histology and Histopathology*.

[9] Choi J., Jung W. H., Koo J. S. (2013). Metabolism-related proteins are differentially expressed according to the molecular subtype of invasive breast cancer defined by surrogate immunohistochemistry. *Pathobiology*.

[10] Kim S., Kim D. H., Jung W.-H., Koo J. S. (2013). Expression of glutamine metabolism-related proteins according to molecular subtype of breast cancer. *Endocrine Related Cancer*.

[11] Jung Y. Y., Lee Y. K., Koo J. S. (2015). Expression of cancer-associated fibroblast-related proteins in adipose stroma of breast cancer. *Tumor Biology*.

[12] Elston C. W., Ellis I. O. (1991). Pathological prognostic factors in breast cancer. I. The value of histological grade in breast cancer: experience from a large study with long-term follow-up. *Histopathology*.

[13] Hammond M. E. H., Hayes D. F., Dowsett M. (2010). American Society of Clinical Oncology/College Of American Pathologists guideline recommendations for immunohistochemical testing of estrogen and progesterone receptors in breast cancer. *Journal of Clinical Oncology*.

[14] Wolff A. C., Hammond M. E., Schwartz J. N. (2007). American Society of Clinical Oncology/College of American Pathologists guideline recommendations for human epidermal growth factor receptor 2 testing in breast cancer. *Journal of Clinical Oncology*.

[15] Henry L. R., Lee H. O., Lee J. S. (2007). Clinical implications of fibroblast activation protein in patients with colon cancer. *Clinical Cancer Research*.

[16] Goldhirsch A., Wood W. C., Coates A. S. (2011). Strategies for subtypes—dealing with the diversity of breast cancer: highlights of the St Gallen International Expert Consensus on the primary therapy of early breast cancer 2011. *Annals of Oncology*.

[17] Marcell Szász A., Lánczky A., Nagy Á. (2016). Cross-validation of survival associated biomarkers in gastric cancer using transcriptomic data of 1,065 patients. *Oncotarget*.

[18] Richardson A. D., Yang C., Osterman A., Smith J. W. (2008). Central carbon metabolism in the progression of mammary carcinoma. *Breast Cancer Research and Treatment*.

[19] Meadows A. L., Kong B., Berdichevsky M. (2008). Metabolic and morphological differences between rapidly proliferating cancerous and normal breast epithelial cells. *Biotechnology Progress*.

[20] Yoon J. K., Kim D. H., Koo J. S. (2014). Implications of differences in expression of sarcosine metabolism-related proteins according to the molecular subtype of breast cancer. *Journal of Translational Medicine*.

[21] Kim S. K., Jung W. H., Koo J. S. (2014). Differential expression of enzymes associated with serine/glycine metabolism in different breast cancer subtypes. *PLoS One*.

[22] Kim S., Lee Y., Koo J. S. (2015). Differential expression of lipid metabolism-related proteins in different breast cancer subtypes. *PLoS One*.

[23] Manandhar S., Choi B.-h., Jung K.-A. (2012). NRF2 inhibition represses ErbB2 signaling in ovarian carcinoma cells: implications for tumor growth retardation and docetaxel sensitivity. *Free Radical Biology & Medicine*.

[24] Ahmad F., Dixit D., Sharma V. (2016). Nrf2-driven TERT regulates pentose phosphate pathway in glioblastoma. *Cell Death & Disease*.

[25] Harami-Papp H., Pongor L. S., Munkácsy G. (2016). TP53 mutation hits energy metabolism and increases glycolysis in breast cancer. *Oncotarget*.

[26] Földi M., Stickeler E., Bau L. (2007). Transketolase protein TKTL1 overexpression: a potential biomarker and therapeutic target in breast cancer. *Oncology Reports*.

[27] Kuhajda F. P., Jenner K., Wood F. D. (1994). Fatty acid synthesis: a potential selective target for antineoplastic therapy. *Proceedings of the National Academy of Sciences of the United States of America*.

[28] Vazquez-Martin A., Colomer R., Brunet J., Lupu R., Menendez J. A. (2008). Overexpression of fatty acid synthase gene activates HER1/HER2 tyrosine kinase receptors in human breast epithelial cells. *Cell Proliferation*.

[29] Jiang P., Du W., Wang X. (2011). p53 regulates biosynthesis through direct inactivation of glucose-6-phosphate dehydrogenase. *Nature Cell Biology*.

[30] Patra K. C., Hay N. (2014). The pentose phosphate pathway and cancer. *Trends in Biochemical Sciences*.

[31] Elf S., Lin R., Xia S. (2017). Targeting 6-phosphogluconate dehydrogenase in the oxidative PPP sensitizes leukemia cells to antimalarial agent dihydroartemisinin. *Oncogene*.

[32] Catanzaro D., Gaude E., Orso G. (2015). Inhibition of glucose-6-phosphate dehydrogenase sensitizes cisplatin-resistant cells to death. *Oncotarget*.

[33] Wang X., Wu G., Cao G. (2015). Zoledronic acid inhibits the pentose phosphate pathway through attenuating the Ras‑TAp73‑G6PD axis in bladder cancer cells. *Molecular Medicine Reports*.

[34] Li L., Fath M. A., Scarbrough P. M., Watson W. H., Spitz D. R. (2015). Combined inhibition of glycolysis, the pentose cycle, and thioredoxin metabolism selectively increases cytotoxicity and oxidative stress in human breast and prostate cancer. *Redox Biology*.

